# Acquired hydrocephalus is associated with neuroinflammation, progenitor loss, and cellular changes in the subventricular zone and periventricular white matter

**DOI:** 10.1186/s12987-022-00313-3

**Published:** 2022-02-22

**Authors:** Maria Garcia-Bonilla, Leandro Castaneyra-Ruiz, Sarah Zwick, Michael Talcott, Ayodamola Otun, Albert M. Isaacs, Diego M. Morales, David D. Limbrick, James P. McAllister

**Affiliations:** 1 Department of Neurosurgery, Washington University in St. Louis School of Medicine, St. Louis, MO 63110 USA; 2 Division of Comparative Medicine, Washington University in St. Louis School of Medicine, St. Louis, MO 63110 USA; 3 Division of Neurosurgery, Department of Clinical Neurosciences, University of Calgary, Alberta, T2N 2T9 Canada

**Keywords:** Kaolin-induced hydrocephalus, Pig model, Ventriculomegaly, Subventricular zone reduction, White matter alteration, Neuroinflammation

## Abstract

**Background:**

Hydrocephalus is a neurological disease with an incidence of 80–125 per 100,000 births in the United States. Neuropathology comprises ventriculomegaly, periventricular white matter (PVWM) alterations, inflammation, and gliosis. We hypothesized that hydrocephalus in a pig model is associated with subventricular and PVWM cellular alterations and neuroinflammation that could mimic the neuropathology described in hydrocephalic infants.

**Methods:**

Hydrocephalus was induced by intracisternal kaolin injections in 35-day old female pigs (n = 7 for tissue analysis, n = 10 for CSF analysis). Age-matched sham controls received saline injections (n = 6). After 19–40 days, MRI scanning was performed to measure the ventricular volume. Stem cell proliferation was studied in the Subventricular Zone (SVZ), and cell death and oligodendrocytes were examined in the PVWM. The neuroinflammatory reaction was studied by quantifying astrocytes and microglial cells in the PVWM, and inflammatory cytokines in the CSF.

**Results:**

The expansion of the ventricles was especially pronounced in the body of the lateral ventricle, where ependymal disruption occurred. PVWM showed a 44% increase in cell death and a 67% reduction of oligodendrocytes. In the SVZ, the number of proliferative cells and oligodendrocyte decreased by 75% and 57% respectively. The decrease of the SVZ area correlated significantly with ventricular volume increase. Neuroinflammation occurred in the hydrocephalic pigs with a significant increase of astrocytes and microglia in the PVWM, and high levels of inflammatory interleukins IL-6 and IL-8 in the CSF.

**Conclusion:**

The induction of acquired hydrocephalus produced alterations in the PVWM, reduced cell proliferation in the SVZ, and neuroinflammation.

**Supplementary Information:**

The online version contains supplementary material available at 10.1186/s12987-022-00313-3.

## Introduction

Hydrocephalus is a neurologic condition resulting from cerebrospinal fluid (CSF) flow impairment that promotes the expansion of the brain ventricles [1–3]. Although its prevalence is 80–125 cases per 100,000 births [4, 5], it is the most common disease treated by pediatric neurosurgeons [2].

The pathophysiology of hydrocephalus includes a variety of alterations in the biochemistry, structure, and physiology of the brain [6, 7]. Ischemia/hypoxia and alterations in the periventricular white matter (PVWM), including myelin destruction and loss of oligodendrocytes progenitors, have been described in human and experimental models [8–11]. Inflammation has also been suggested to play a role in the pathogenesis of acquired hydrocephalus [12] through the increase of microglial and astroglial reactions [6, 9, 13, 14]. In addition, the levels of pro-inflammatory molecules, such as interleukins IL1α, IL4, IL6, IL12, Tumor Necrosis Factor-alpha (TNF-α), or Transforming Growth Factor-beta, some of them activated by the Toll Like Receptor 4 downstream pathway [15], in the CSF correlate with the severity of the disease [8, 12, 16–23].

The subventricular zone (SVZ), which is one of the two regions of the mature brain where neural stem cells reside [24, 25], is altered in hydrocephalus [26–30]. The SVZ comprises different types of cells: type A (neuroblasts), B1 and B2 (astroglial characteristics), C (intermediate progenitors), and E (ependyma, with no proliferative capacity) [25, 31]. These proliferative layers of progenitors can generate both neurons and glia, including oligodendrocytes [25, 32, 33]. In hydrocephalus, SVZ progenitors have been shown to be reduced or altered in humans [30], ferret model [27], and rodent models [26, 28, 29, 34].

A commonly used method for inducing experimental hydrocephalus is the injection of kaolin (aluminum silicate) into the cisterna magna [27, 35–40], which obstructs CSF flow as a result of a local inflammatory reaction and fibrous scarring [7, 35, 36]. This method has been developed mostly in non-gyrencephalic animals [41]. However, there is increasing need for models utilizing large animals with gyrencephalic brains to test the efficacy of new hydrocephalus treatments in preclinical studies [27]. The domestic pig (*Sus scrofa domesticus*) is an appropriate model to study human brain diseases, as it has several anatomical and physiological similarities to humans [42–44]. Also, the larger size of pig brains than other relatively animal models makes it feasible to perform current neurosurgical treatments for human hydrocephalus [45–48].

The present investigation aimed to characterize the neuropathology in a pig model of acquired hydrocephalus by analyzing the proliferative cells in the SVZ, PVWM alterations, and markers of neuroinflammation in the brain parenchyma and CSF. Further, this study elucidates the neuropathogenesis of acquired hydrocephalus in a pig model as the first step in evaluating the long-term biological sequelae of clinical hydrocephalus treatments, including endoscopic third ventriculostomy with choroid plexus cauterization (ETV-CPC) and CSF shunting.

## Materials and methods

### Experimental design

Hydrocephalus was induced in juvenile female pigs (*Sus scrofa domesticus*), aged 33–43 days old, via a kaolin injection. Females were used because they exhibit more exploratory behavior than males [49], and thus were more amenable to the cognitive assessments we have reported previously [50]. Animals were separated into two cohorts: (1) to study the neuropathology in the tissue (hydro group, n = 7; and sham control group n = 6), and (2) to analyze inflammation in the CSF (hydro group, n = 10; sham control group, n = 6). As controls, the group of age-matched pigs was administered with sterile saline (sham-injected control group). Pigs were sacrificed 19–40 days after the induction of hydrocephalus or the saline injection. There were three additional cases of pigs injected with kaolin that did not develop hydrocephalus after 12–48 days of induction (n = 3). Ventriculomegaly was determined as 2 standard desviation from the sham control volume mean[50]. These “kaolin-injected, non-hydrocephalic” pigs were used to analyze the microglial reaction cause by hydrocephalus, and not by the kaolin per se.

### Animals

Pigs were obtained from Oak Hill Genetics LLC (university-approved vendor, Ewing, IL, USA) and housed at the Washington University in St. Louis vivarium in standard pens with raised flooring, fed with a standard pig chow (Purina Porcine Grower Diet 5084), and provided access to water ad libitum. Before any procedure, the pigs were quarantined for at least 3 days and allowed to acclimate to the facility, and their normal health and behavior were confirmed by the institutional veterinary staff.

The design of the experiments, housing, handling, care, surgery, and pre/post-operative management of the animals were approved by the Washington University Institutional Animal Care and Use Committee and performed in an AAALAC-accredited facility in compliance with the Guide for the Care and Use of Laboratory Animals and the Animal Welfare Act.

### Induction of hydrocephalus

Pigs were sedated, intubated, and anesthetized using 2.5% isoflurane in O_2_/N_2_O. The dorsum of the neck was shaved, sterilized with Povidone iodine and 70% alcohol. Using sterile technique, an 18-gauge spinal needle attached to an extender, and a 3 mL syringe were used to access the cisterna magna. After the collection of 0.5–1 mL of CSF, 1.3 mL of sterile 25% kaolin (K2502, Aqua Solutions, Deer Park, TX, US) suspension was injected slowly into the cisterna magna. Sham controls received similar injections of sterile saline.

Animals were monitored post induction while recovering from the anesthetic and observed for signs of fever, pain and neurological impairment (locomotor or sensorimotor behavior impairment, ataxia, imbalance, loss of alertness) and ability to eat and drink every 4 h for the next 12–15 h, and every day after that for 10 days. They received subcutaneous injections of buprenorphine (0.12–0.24 mg/kg) or carprofen (2–4 mg/kg) analgesic to reduce potential pain, and acetaminophen (162.5 mg) to reduce fever if needed. These analgesics were administered on a case-by-case basis depending on clinical signs and symptoms.

### Magnetic resonance imaging

Magnetic resonance imaging (MRI) was performed on the pigs after the induction of hydrocephalus or the saline injection at sacrifice (19–40 days post induction). The pigs were sedated as described above and maintained at 2.5% isoflurane in O_2_/N_2_O. Respiration and external body temperature were monitored every 5 min during imaging. The images were attained with Siemens Prisma 3.0-Tesla MR scanner with a 60-cm bore diameter, a 20-channel head coil, an 80 mT/m gradient field, and a slew rate of 200 mT/ms. T1- and T2-weighted images (0.9 mm of slice thickness) were obtained with a 3D fast spin-echo sequence with an echo train length of 8, Field of view 205 × 205 mm (256 × 256 voxels), and a voxel size of 0.8 mm. T1- and T2-wighted magnetization-prepared rapid acquisition with gradient echo (mprage) scan time varied from 4 to 11 min (T1: Repetition time (TR) 2300 ms, Time to Echo (TE) 2.36 ms, 2 averages; T2: TR 3200 ms, TE 409 ms, 2 averages).

### Ventricular volume analysis and definition of the frontal horn and body of the lateral ventricles

Three-dimensional reconstruction and analysis of ventricular volumes were performed using the sagittal, axial, and coronal MRI images (0.9 mm) and the software ITK-SNAP v3.8.0 (http://www.itksnap.org/pmwiki/pmwiki.php, University of Pennsylvania, US). The ventricles were segmented, and the volume was calculated in mm^3^ using the volumes and statistics tool of the software. Lateral ventricles were divided into the four main regions as described in Scelsi et al. [51]: (1) the frontal horn, from the anterior wall to the foramen of Monro; (2) the body, from the foramen of Monro to the point where the septum pellucidum ends and the corpus callosum and fornix meet (hippocampal commissure); (3) the atrium, a triangular cavity that opens anteriorly into the body and basely into the temporal horn; and (4) temporal horn, that extends anteriorly from the atrium below the thalamus and terminates at the amygdala.

### Histology and immunofluorescence

Control and hydrocephalic pigs were sedated and intravenous heparin (150 mg/kg) was administered to reduce risk of clotting in brain before the perfusion. After 5 min, they were sacrificed under anesthesia with intravenous sodium pentobarbital (120 mg/kg), and transcardially perfused with Phosphate Buffered Saline (PBS, 0.1 M pH 7.2) followed by 4% paraformaldehyde diluted in the same buffer. Fixed brains were dissected and postfixed in the same solution for 48 h at room temperature.

Fixed brains were embedded in paraffin and sectioned serially at a thickness of 10 µm. After heat-induced antigen retrieval in citrate (50 mM, pH 6.0), primary antibodies (Table 1) were incubated for 18 h at 22 °C. Secondary antibodies conjugated with Alexa Fluor 488 or Alexa Fluor 555 (RRID: AB_2576217, AB_2535844, AB_2534069, Thermo Fisher, Waltham, MA) were applied for 1 h at room temperature, dilution 1:500. 4′,6-Diamidino-2-phenylindole dihydrochloride (DAPI, Molecular probes Life technology, D1306, RRID:AB_2629482) was used for nuclear staining, dilution 1:5000, 5 min. The antibodies were diluted in PBS containing 0.1% Triton X-100 (Sigma). For negative controls, primary antibodies were not added.

**Table 1:** Primary antibodies

Antibody	Manufacturer	Catalog number	Research resource identifier (RRID)	Dilution	Host
βIII tubulin	Promega	G712A	AB_430874	1:500	Mouse
βIV tubulin	Abcam	ab11315	AB_297919	1:50	Mouse
Cleaved Caspase-3	Cell Signaling Technology	9661	AB_2341188	1:400	Rabbit
GFAP	Abcam	ab7260	AB_305808	1:400	Rabbit
GFAP	Sigma	C9205	AB_476889	1:1000	Mouse
Iba1	Wako (Fisher Scientific)	NC9288364	AB_839504	1:500	Rabbit
Ki67	Abcam	Ab15580	AB_443209	1:500	Rabbit
Olig2	EMD Millipore	ab9610	AB_570666	1:200	Rabbit
N-cadherin	Invitrogen	33-3900	AB_2313779	1:25	Mouse
PCNA	Abcam	ab29	AB_303394	1:500	Mouse
Vimentin	Abcam	ab92547	AB_10562134	1:200	Rabbit

### Hematoxylin–eosin staining

Paraffin sections were deparaffinized and hydrated. Hematoxylin (Harris, Biocare Medical, CA, US, 061920A-2) was applied for 7 min, rinsed for 5 min in running tap water, and differentiated by a fast dip in 0.5% acid ethanol. Then, they were stained with 0.5% eosin (Sigma, 1.09844.1000) for 2 min, dehydrated, and mounted with xylene based mounting medium.

### Cytokine and chemokine analysis

To study the immune response in the CSF, the Porcine Cytokine & Chemokine 9-Plex ProcartaPlex Panel 1 was used (Thermo Fisher, EPX090-60829-901, RRID:AB_2576098). This immunoassay is based on the principles of a sandwich ELISA and enables the study of the immune response through the analysis of 9 protein targets in a single well: interferon (IFN) alpha, IFN gamma, IL1 beta, IL10, IL12/IL-23p40, IL4, IL6, IL8 (CXCL8), and TNF-alpha. CSF samples were centrifuged at 2000*g* to remove the cell debris, and 50 µL of the supernatant were analyzed in each well following the manufacturer’s instructions. In this approach, CSF before the kaolin injection was used as baseline (*pre*) for comparison to the CSF after the kaolin administration (*post*) (n = 10). Also, CSF before (pre-induction) and 30 days after (post-induction) the sham control injections (n = 6) was analyzed to study the possible effect of the injection of saline into the cisterna magna. Duplicates were run for each sample.

### Image analysis and quantification

Immunofluorescence images (1024 × 1024-pixel resolution) were obtained with a Zeiss LSM 880 Airyscan Two-Photon Confocal Microscope (Oberkochen, Germany). Bright-field and fluorescent micrographs were obtained with a Leica CTR5500 fluorescent microscope (Leica, Wetzlar, Germany).

For each experiment, images were obtained in batches using the same settings. Figures were composed using Adobe Photoshop CS5.1 (Adobe Inc., San Jose, CA, US), and the same minimal changes in brightness and contrast were applied. The amount of ependymal disruption (βIV tubulin and glial fibrillary acidic protein- GFAP stainings) was estimated in the entire lateral wall of the frontal horn and body of the lateral ventricles in one paraffin section per animal. The results are shown in the percentage of disrupted areas to the total ventricular area. The percentage of the area occupied by astrocytes (GFAP+ cells) and vimentin + cells in PVWM and SVZ were quantified per area, as the immunolabeling did not allow for the quantification of the number of cells. The cell densities of Iba1 + , proliferating cell nuclear antigen (PCNA) + , Ki67 + , GFAP + (with no expression of vimentin), βIII tubulin + , caspase-3 + , Olig2 + cells, and SVZ area were calculated in 3 fields of 3 different areas in 2 parietal cortex sections per animal. The ependyma was not included in these quantifications. The SVZ area was analyzed in 3 parietal sections every 50 µm approximately. Finally, the cytokine profile in the CSF was analyzed in two replicates per animal.

### Three-dimensional reconstruction and analysis of the microglial cells

Microglia morphology analysis was performed as described in the literature [52, 53]. Sections were immunostained with anti-Iba1 as described above. Imaging was performed on the Zeiss LSM 880 microscope using a 20× 0.8 NA objective using the same settings with a resolution of 1024 × 1024 pixels. 1-μm steps in z-direction were used to do Z-stacks. Images were analysed using IMARIS software (Bitplane, Concord, MA, US). Tracing was performed in a region of interest comprising one cell. The ramification density and arborization were analyzed for microglia as their morphology is considered a direct indicator of their activity. Thus, cells were eligible for analysis only when the entire cell was labeled and all processes were visible in the 3D reconstruction. The automatic detection mode and the same configuration were used for all the samples. Three microglial cells from three fields of the PVWM were reconstructed per animal. The equivalent area of the PVWM adjacent to the frontal horn and body of the lateral ventricles was chosen in each animal per group.

### Statistical analysis

Statistical analyses were performed using KaleidaGraph (Synergy Software, Reading, PA, USA) and GraphPad Software (San Diego, CA, USA). Animals were numbered without indication of the group. All values are reported in the figures as mean ± SEM with a 95% confidence interval. The Wilcoxon–Mann–Whitney test and Student’s t-test were applied for hypothesis testing in situations requiring non-parametric and parametric analyses, respectively (shown with * in the graphs in the figures). A one-way ANOVA test was performed to confirm the differences found between the sham control group and pre/post-induction of hydrocephalus groups in the ELISA analyses. Simple linear regression analysis was performed to model the relationship between the SVZ area, CSF cytokines and total ventricular volumes, and astrocytes, microglia and frontal horn/body volumes in the hydrocephalic pigs. When the *F* probability from Student’s t-test was < 0.05, the variance was considered unequal. *P* < 0.05 based on both tests was considered statistically significant.

## Results

### Hydrocephalus in the juvenile pig model

**Fig. 1:**
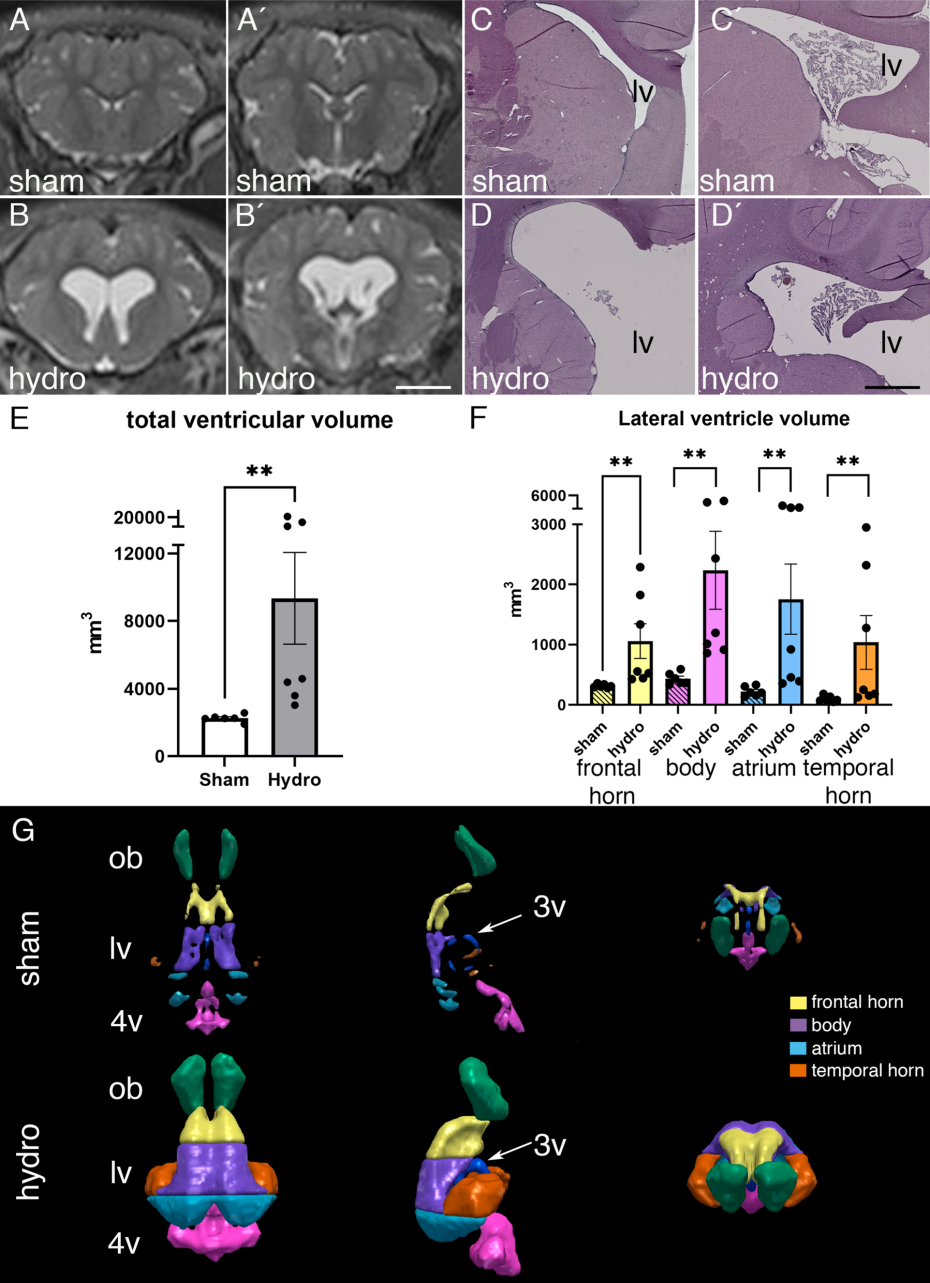
The injection of kaolin induced hydrocephalus in pigs. Representative magnetic resonance images showing the most pronounced increase of the body (**A′**, **B′**) compared to the frontal horn (**A**, **B**) of the lateral ventricles from sham control and hydrocephalic pigs. Representative images of hematoxylin–eosin staining in (**C**, **C′**) sham control, and (**D**, **D′**) hydrocephalic pigs showing the most expansion of the lateral ventricles (*lv*). Scale bars represent 1 mm in **A**–**B′**, and 0.5 mm in **C–D′**. **E** Graph illustrating the significant increase in total ventricular volume in hydrocephalic pigs. **F** Graph illustrating the proportional volume changes in the frontal horn and body; note that while both regions exhibited significant ventriculomegaly, the body expanded more than the frontal horn. **G** Representative 3D reconstruction of the ventricular system, based on ventricular volumes and including the olfactory ventricle (*ob*, green), frontal horn (yellow), body (purple), atrium (light blue), temporal horn (orange), third ventricle (dark blue and *3v* with arrow), and forth ventricle (*4v*, pink) in the sham control (*sham*) and hydrocephalic (*hydro*) pig. Means with 95% confidence intervals are shown. **p = 0.001 Wilcoxon–Mann–Whitney test

Administration of kaolin into the cisterna magna produced ventriculomegaly in the pigs 19–40 days after the induction. MRI revealed the dilatation of all portions of the cerebral ventricles, especially the lateral ventricles (Fig. 1A, B, Additional file 1: Fig. S1). Ventricular dilation was also confirmed by the histopathological analysis using hematoxylin–eosin staining (Fig. 1C, D). The expansion of the ventricles was approximately eight times larger in the hydrocephalic pigs (9340.3 ± 2716.9 mm^3^) than the sham control pigs (2251 ± 86.8 mm^3^) (mean ± SEM*, p* = 0.001, Wilcoxon–Mann–Whitney Test) (Fig. 1E).

The ventricular volumes of four areas of the lateral ventricles were obtained separately (Fig. 1F, G). 3D ventricular volume measurements showed that the most expanded region was the body, which was almost five times larger (2237 ± 649 mm^3^) in the hydrocephalic animals compared to the sham controls (438.5 ± 40.7 mm^3^) (mean ± SEM*, p* = 0.001, Wilcoxon–Mann–Whitney Test) (Fig. 1F).

The pathology associated with hydrocephalus in the pigs was studied by analyzing the SVZ, the neuroinflammatory reaction in the brain parenchyma and CSF, and alterations in the PVWM adjacent to both frontal horn and body of the lateral ventricles.

### Ependymal disruption of the ventricular walls of the hydrocephalic pigs

**Fig. 2:**
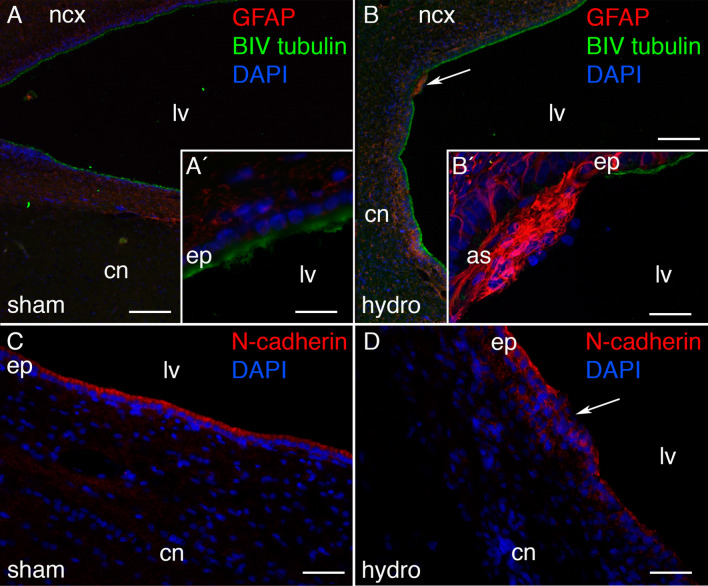
Ependymal disruption in the hydrocephalic pigs. Ependymal disruptions were detected in the hydrocephalic pigs through labeling the astrocytes (GFAP, fluorescence in *red*) and ependymal cells (βIV tubulin, *green*) in the periventricular area from **A**, **A′** a sham control, and **B** a hydrocephalic pig. **B′** Magnification of the top area pointed in **B** showing the ventricular zone disruption. **C**, **D** N-cadherin labelling also confirmed the disruption in the ventricular zone in the hydrocephalic pigs. Nuclear staining with DAPI in *blue*. Scale bars represent 120 µm in **A**, **B**; 25 µm in **A′**, and 40 µm in **B′**. Images were obtained under the confocal microscope, and a Z-stack of 10 µm was composed with ImageJ software. *as* astrocyte reaction, *cn* caudate nucleus, *ep* ependymal, *ncx* neocortex

Ependymal disruption or loss, as a common pathology associated with hydrocephalus in vivo and in vitro [29, 54–58], was studied in kaolin-injected animals. Whereas in the sham control pigs the lateral ventricle walls were found completely covered by healthy ependymal cells (Fig. 2A, C), kaolin-injected pigs showed disrupted areas (Fig. 2B, D). In these areas, ependyma, labeled with βIV tubulin and N-cadherin, was replaced by reactive astrocytes (GFAP + cells). Ependymal disruption, estimated in the entire cell layer lining the frontal horn and body of the lateral ventricles, extended for 5.46 ± 1.8 µm^2^ and 12.89 ± 5.5 µm^2^ (mean ± SEM), respectively, in both parts of the lateral ventricles. These disrupted areas did not follow a specific pattern, and no particular population of ependymocytes was particularly affected. No disruption occurred in the ependymal layer overlying the SVZ.

### Alterations in the periventricular white matter of the hydrocephalic pigs

**Fig. 3:**
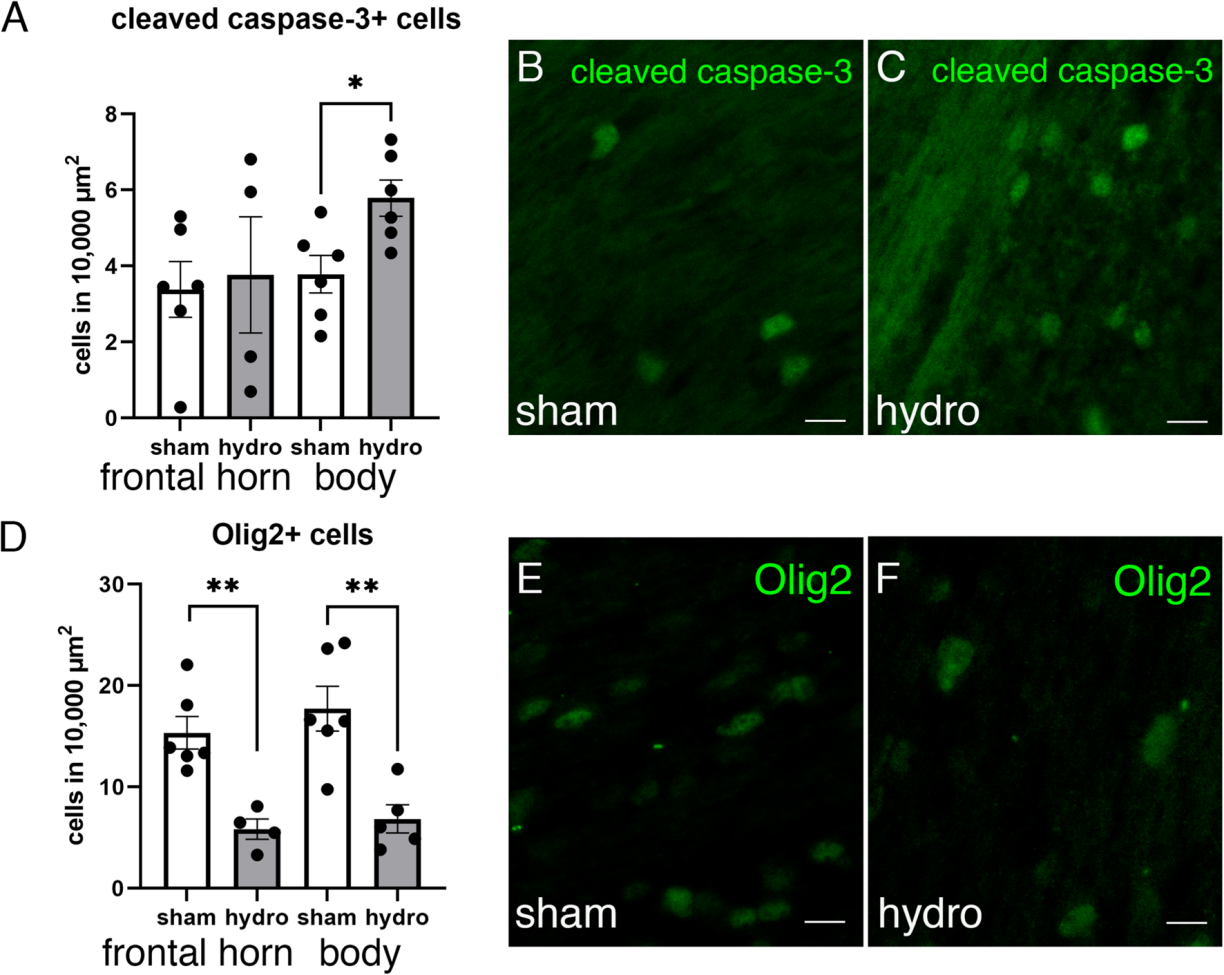
Increase of cell death and reduction of oligodendrocytes in the PVWM after induction of hydrocephalus. **A** The number of positive cells for cleaved caspase-3, which labels cell death, was found to increase in the PVWM adjacent to the body of the lateral ventricles in hydrocephalic (*hydro*) pigs compared with sham control (*sham*) pigs. **B**, **C** Representative images illustrating the increase of cell death in the PVWM adjacent to the body of the hydrocephalic pigs. Magnifications are the same. **D** In addition, the hydrocephalic pigs showed a reduction of Olig2+ cell density in the PVWM from both areas adjacent to the lateral ventricles. **E**, **F** Representative images used for quantification of Olig2+ in sections from both groups showing the decrease of oligodendrocytes in the PVWM adjacent to the body of the hydrocephalic pigs. Magnifications are the same. Scale bars represent 20 µm for caspase-3 and 10 µm for Olig2. Images were obtained under the fluorescent microscope. Means with 95% confidence intervals are shown. *p = 0.026; **p = 0.0095 in the frontal horn and **p = 0.0087 in the body, Wilcoxon–Mann–Whitney test

The PVWM adjacent to the body of the lateral ventricles showed alterations after the induction of hydrocephalus. Interestingly, the PVWM adjacent to the body in the kaolin-injected pigs presented a 44% increase in the number of apoptotic cells (cleaved caspase-3 positive cells) compared to the sham controls (*p* = 0.026 Wilcoxon–Mann–Whitney Test) (Fig. 3A–C). Additionally, the number of Olig2 + cells, which label both progenitor and mature oligodendrocytes [33], showed a 67% reduction in the PVWM of the hydrocephalic animals adjacent to the frontal horn (*p* = 0.0095 Wilcoxon–Mann–Whitney Test) and body (*p* = 0.0087 Wilcoxon–Mann–Whitney Test) (Fig. 3D–F).

### Cell proliferation and oligodendrocytes in the SVZ in the hydrocephalic condition

**Fig. 4:**
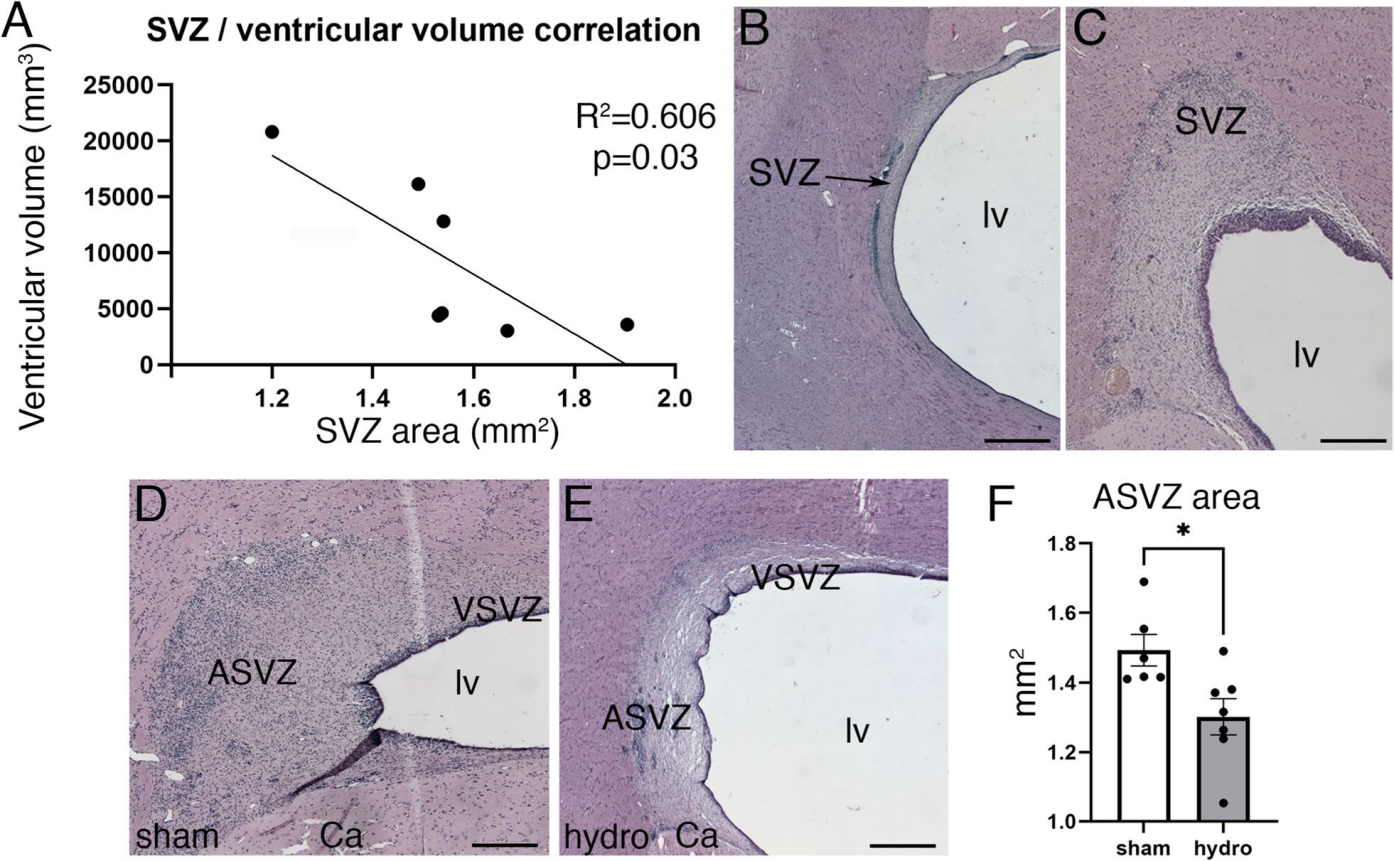
Correlation between the decrease of subventricular zone area and ventricular volume in the hydrocephalic pigs. **A** The increase of ventricular volumes correlated with the decrease of the SVZ area in the hydrocephalic pigs. **B**, **C** Hematoxylin–eosin staining showing the decrease of the SVZ area when the ventricular volume increased. The SVZ was divided in its two main areas, the abventricular SVZ (AVSZ) and ventricular SVZ (VSVZ) in **D** sham control pig and **E** hydrocephalic pig. **F** The AVSZ area decreased in the hydrocephalic pigs. Scale bars represent 300 µm in **B**, **C**; 250 µm in **D**, **E**. *p = 0.014, Wilcoxon–Mann–Whitney test. Simple linear regression with a R squared of 0.6065, p-value 0.03. Images were obtained under the bright microscope. *lv* lateral ventricle lumen

The area of the SVZ was found to decrease and correlate (F = 7.708, R^2^ = 0.6065, p = 0.039, Simple linear regression) to the ventricular volume increase in the hydrocephalic cases (Fig. 4A–C). As described by Costine et al. [59], the SVZ can be divided into two different areas, one close to the lateral ventricle (ventricular SVZ—VSVZ) and the other deep to the VSVZ, named the abventricular SVZ (ASVZ). In the present investigation, we focused our analyses on the ASVZ, but further studies will analyze the VSVZ.

**Fig. 5:**
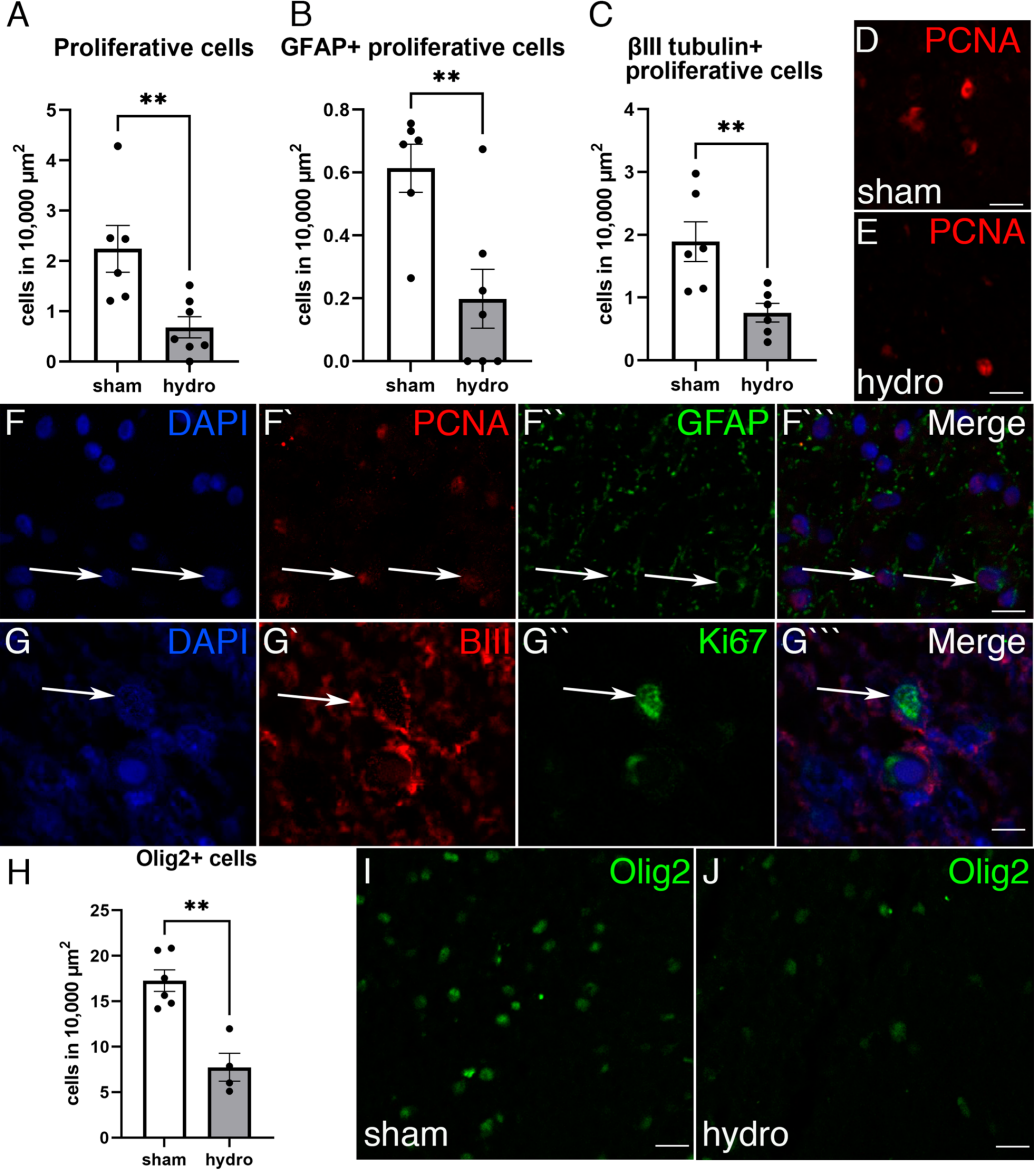
Subventricular zone alteration in the hydrocephalic pigs. The cell densities of **A** proliferative cells (PCNA+), **B** PCNA+ with GFAP+ , and **C** βIII tubulin + Ki67 + cells in the ASVZ were reduced in the hydrocephalic (*hydro*) pigs compared with the sham control (*sham*) pigs. Representative immunofluorescence for PCNA (*red*) in sections of the ASVZ from **D** a sham control and **E** a hydrocephalic pig illustrating the decrease of proliferative cells. Magnifications are the same. Separated channels for fluorescence for (**F**–**F′′′**) PCNA (*red*) and GFAP (*green*), and (**G**–**G′′′**) βIII tubulin (*red*) and Ki67 (*green*) with same magnifications. **H** Oligodendrocytes (Olig2, *green*) were also reduced in the ASVZ of **J** hydrocephalic pigs compared with the **I** sham control. Nuclear staining in *blue* with DAPI. Scale bars represent 15 µm in **D**, **E**; 15 µm in **F–F′′′**; 12 µm in **G–G′′′,** 30 µm in **I**, **J**. Images were obtained under the confocal microscope with 1 µm thickness. **p = 0.0047 for PCNA, **p = 0.0082 for PCNA + GFAP, **p = 0.0087 for βIII tubulin + Ki67, **p = 0.0095 for Olig2, Wilcoxon–Mann–Whitney test

In the ASVZ, sham control pigs showed a larger area compared to hydrocephalic pigs (*p* = 0.014 Wilcoxon–Mann–Whitney Test) (Fig. 4D–F). The neurogenic niche in the ASVZ was found affected in the kaolin-injected pigs (Fig. 5). The cell proliferation (labelled with PCNA and Ki67) in the ASVZ was decreased by 75% in the hydrocephalic condition compared with the sham control (*p* = 0.0047 Wilcoxon–Mann–Whitney Test) (Fig. 5A, D, E). Double positive cells for PCNA and GFAP with no expression of vimentin (and excluding the ependyma from the quantification), immunostaining mostly the type B cells of the SVZ (astrocytes and progenitor cells) [31], were also decreased in the ASVZ of the hydrocephalic condition (*p* = 0.0082, Wilcoxon–Mann–Whitney Test) (Fig. 5B, F). Additionally, type A cells (neuroblasts), labelled with βIII tubulin and Ki67, decreased in the ASVZ of the hydrocephalic pigs by 40.1% (*p* = 0.0087, Wilcoxon–Mann–Whitney Test) (Fig. 5C, G). However, the total number of GFAP + and vimentin + cells [31] present in the ASVZ was not significantly decreased in the hydrocephalic pigs when compared with the sham control pigs (Additional file 2: Fig. S2). In addition, the number of the oligodendrocyte progenitors (Olig2+) was also 57% lower after the kaolin injection in the ASVZ (*p* = 0.0095 Wilcoxon–Mann–Whitney Test) (Fig. 5H–J).

### Astrocytes and microglia in the PVWM adjacent to the body of the lateral ventricles from the hydrocephalic pigs

**Fig. 6:**
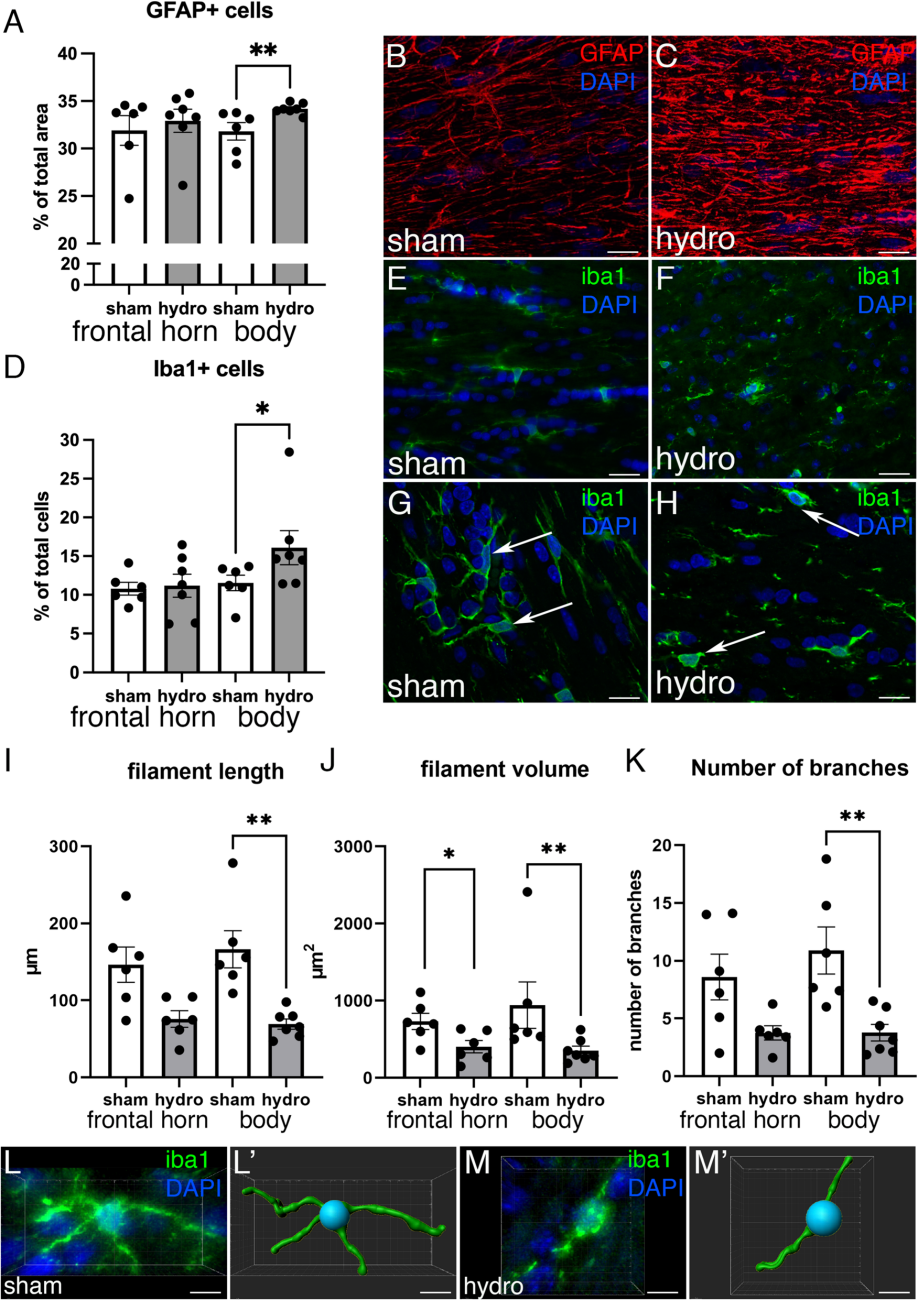
Analysis of the astroglia and microglia reaction in the PVWM after the induction of hydrocephalus. **A** Density of GFAP+ cells (percentage of the area occupied by astrocytes respect to the total measured area) was increased in the PVWM adjacent to the body of the lateral ventricles, but not in the frontal horn, in the hydrocephalic pigs. Representative images used for quantification for GFAP labeling (*red*) in paraffin sections of the PVWM from **B** a sham control and **C** a hydrocephalic pigs. Magnifications are the same. **D** Density of Iba1+ cells (percentage of microglial cells per total number of cells) was also increased in the PVWM adjacent to the body of the lateral ventricles in hydrocephalic pigs. Representative images used for quantification for Iba1 labeling (*green*) in the PVWM from **E** sham control and **F** hydrocephalic pigs. Magnifications are the same. Microglia was reactive in the hydrocephalic condition as illustrated in the details of **G** healthy Iba1+ cells (*green*) in a sham pig, and **H** in a hydrocephalic animal (*white arrows*). The filament length (**I**), volume (**J**), and number of branches (**K**) were reduced in the hydrocephalic pigs. Representative images from an immunofluorescence and its 3D reconstruction of a microglial cells from the PVWM adjacent to the body in a sham control (**L**, **L′**) and a hydrocephalic pig (**M**, **M′**). Scale bars represent 30 µm in **B**, **C**, **E**, **F**; 15 µm in **G**, **H**; and 8 µm in **L**–**M′**. Images were obtained under the confocal microscope, and a Z-stack of 10 µm was composed with ImageJ software in **B**, **C**, **E**, **F**. 1-µm thickness images are shown in **G** and **H**. **p = 0.035 for GFAP, *p = 0.035 for Iba1, **p = 0.0012 filament length, *p = 0.0411 **p = 0.0082 filament volume, **p = 0.0029 number of branches in the analysis of Iba1 morphology, Wilcoxon–Mann–Whitney test

Astrocytes (GFAP+ cells) and microglia (Iba1+ cells) were quantified in the PVWM surrounding the frontal horn and body of the lateral ventricles (Fig. 6). These areas were chosen because of its proximity to the SVZ in the first case, and its largest expansion in the second case. Interestingly, astrocytes were found to increase by 7.2% (Fig. 6A–C) and microglia by 44% (Fig. 6D–F) in the PVWM adjacent to the body (*p* = 0.0035 and *p* = 0.035, Wilcoxon–Mann–Whitney Test, respectively), but not in the frontal horn, in the hydrocephalic pigs compared to the sham control pigs. To understand these differences in the PVWM adjacent to between the frontal horn and body concerning neuroinflammation signs, the total ventricular volume was correlated with the increase of astrocytes and microglia (Additional file 3: Fig. S3). Trend level relationships (not significant) were observed when examining the increase of Iba1 + in the PVWM of the body (Additional file 3: Fig. S3).

In addition to the increase in the number of microglial cells, these cells showed changes in their morphology after the induction of hydrocephalus. The 3D reconstruction preliminary measurements revealed that microglia in the PVWM adjacent to the body displayed 61.2% decrease of filament length (*p* = 0.0012, Wilcoxon–Mann–Whitney Test) 69.8% of volume (*p* = 0.0411 in the frontal horn and *p* = 0.0082, Wilcoxon–Mann–Whitney Test), and 66.7% of number of branches (*p* = 0.0029, Wilcoxon–Mann–Whitney Test) in the hydrocephalic pigs (Fig. 6G–M). The proliferation of the microglia because of hydrocephalus, and not by the kaolin per se, was analyzed in the “kaolin-injected, non-hydrocephalic” pigs. These pigs were injected with kaolin but did not develop ventriculomegaly (Additional file 4: Fig. S4). Interestingly, the kaolin-injected, non-hydrocephalic pigs showed a similar microglial reaction compared with the sham control pigs (Additional file 4: Fig. S4).

No correlations were found with the time following hydrocephalus induction (days post-induction; Additional file 5: Fig. S5) or age at sacrifice (data not shown) and ventricular volume/histology (except days post-induction versus cell death in the PVWM adjacent to the body of the lateral ventricles R^2^ = 0.98, p = 0.0008).

### Neuroinflammatory cytokine and interleukin profile in the CSF from the hydrocephalic pigs

Since neuroinflammation, through the increase of the astrocyte and microglia reaction, was detected in the brain parenchyma, nine key neuroinflammatory markers in the CSF were studied.

**Fig. 7:**
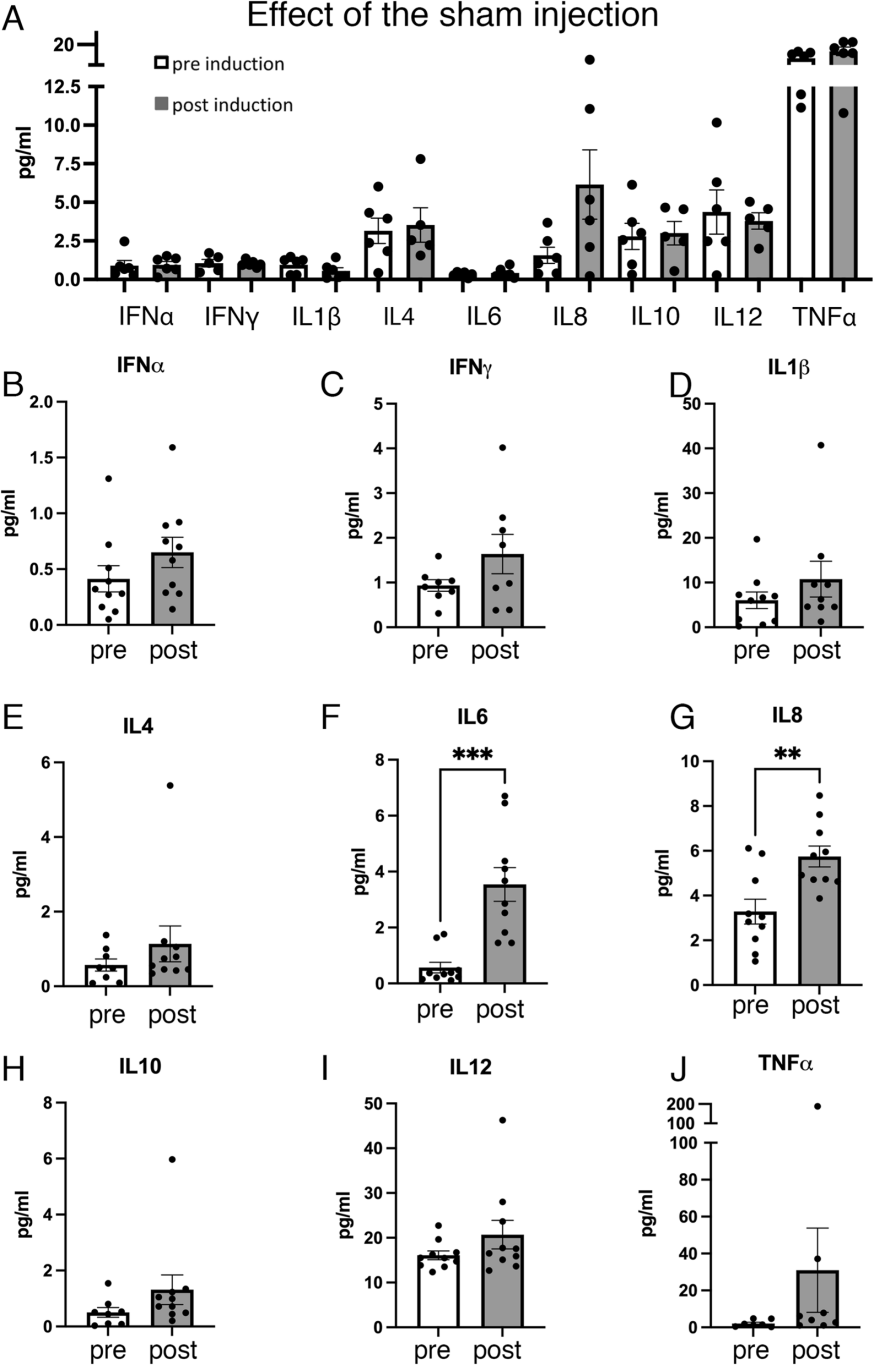
Presence of inflammatory cytokines and interleukins in the cerebrospinal fluid of hydrocephalic pigs. **A** Analysis of the cytokines and interleukins showing no effect of the injection in the cisterna magna in the sham control pigs before (*pre-induction*) and after (*post-induction*) the saline injection. These results discard the hypothesis that surgery or the administration of saline serum into the cisterna magna can cause any changes in the levels of these inflammatory proteins. Analysis in the hydrocephalic pigs of the levels of **B** interferon alpha (IFNα), **C** interferon gamma (IFNγ), **D** interleukin 1 beta (IL1β), **E** interleukin 4 (IL4), **F** interleukin 6 (IL6), **G** interleukin 8 (IL8), **H** interleukin 10, **I** interleukin 12 (IL12), and **J** tumor necrosis factor alpha (TNFα). Neuroinflammation was present since the cytokines/interleukins levels were higher after (*post*) the kaolin administration compared with before the induction (*pre*). Means with 95% confidence intervals are shown. ***p = 0.0002 t = − 4.7 F = 9.818 Student’s t-test for IL-6; **p = 0.0032 t = − 3.4053 F = 1.397 Student’s t-test for IL-8

Firstly, CSF from sham control animals was analyzed. When comparing before (pre-induction) and after (30 days post-sham induction) the administration of saline, none of the cytokines/interleukins were found to increase (Fig. 7A). However, in comparing CSF neuroinflammatory proteins before and after the induction of hydrocephalus, the levels of the interleukins IL6 and IL8 were found to significantly increase (*p* = 0.0002, t = − 4.7, F = 9.818 Student’s t-test for IL6; *p* = 0.0032, t = − 3.405, F = 1.397 Student’s t-test for IL8) (Fig. 7F, G). ANOVA analysis also showed a significant increase of IL8 (F (2, 22) = 7.413, p = 0.0035) when comparing the sham control group with the hydrocephalic animals (post-induction) (p = 0.0149, 95% CI of diff. − 4.993 to − 0.4995), and before and after the induction of hydrocephalus within the same animals (p = 0.0074, 95% CI of diff. − 4.298 to − 0.6297). There were no statistically significant differences between the sham control group and the pre-induction group (p = 0.9468, 95% CI of diff. − 2.429 to 1.965). Also, increased trends of IFNα, IFNγ, IL1β, IL4, IL10, IL12, and TNFα were found in the CSF post kaolin (Fig. 7).

The levels of these proteins were also examined within the same animal before and after the kaolin injection (Additional file 6: Fig. S6). The levels of these nine immune system proteins were increased in almost all hydrocephalic cases after the induction of the disease. Although no significant correlations were found, most protein level increases were associated with an increase in ventricular volume (Additional file 7: Fig. S7).

## Discussion

The present investigation aimed to characterize the periventricular and SVZ in a juvenile pig model of acquired hydrocephalus. Our results showed that the PVWM and the ASVZ exhibited an increase in cell death and reduction of oligodendrocytes and proliferative progenitors. Neuroinflammation was detected in the brain parenchyma and CSF through an increase in the number of astrocytes and reactive microglia in the PVWM, and high levels of inflammatory interleukins IL6 and IL8 in the CSF.

Kaolin injection into the cisterna magna to induce hydrocephalus is a well-characterized technique that has been used in several animal models, including rodents and large animals [20, 27, 35, 37, 39, 40, 60–64]. Its use has the advantage of being a less invasive, inexpensive, and consistent technique to produce ventriculomegaly [36]. Although some variability across age groups was present in the hydrocephalic animals, no correlations were found with ventricular volume, ventricular zone disruption, oligodendrocytes, cell proliferation in the ASVZ, or inflammation, except for cell death in the PVWM adjacent to the body of the lateral ventricles. Thus, these results suggest that no correlation is dependent on time post-induction/age at sacrifice. The development of this model of hydrocephalus in large gyrencephalic animals is clinically relevant because the physical size and expansion of the ventricles makes it feasible to perform neurosurgical treatments, such as insertion of ventricular shunts or ETV-CPC.

In the induced pigs, ventriculomegaly was prominent throughout the whole ventricular system, with communication between the ventricles but restricted flow into the subarachnoid space [50]. The expansion of the ventricles was especially pronunced in the body of the lateral ventricles. Other studies in posthemorrhagic hydrocephalus and fetal ventriculomegaly have reported that the body dilates to a greater extent than other ventricle regions, such as the frontal horn [65, 66].

The induction of hydrocephalus was associated with alterations in PVWM as described in other species including humans [67–70], rodents [8, 20, 35], ferrets [27], and cats [39, 40]. It has been reported that oligodendrocytes present in the PVWM are the most vulnerable in the early stages of hydrocephalus and undergo apoptosis [9, 27]. Our results support that idea since we observed increased cell death, whereas the number of oligodendrocytes decreased after the induction of hydrocephalus. These changes were detected in the PVWM adjacent to the body of the lateral ventricles; thus, the multifactorial mechanism of stretch, compression, edema, or hypoxia may be playing an important role in the myelin destruction [9]. Futures studies, including cognitive testing [49, 50] and diffusion MRI, will be carried out to elucidate the consequences of the PVWM damages.

Neural stem cells are found in two regions of the brain: the SVZ by the dorsolateral wall of the lateral ventricles and the subgranular zone of the dentate gyrus in the hippocampus [24]. In the present study, we have used PCNA, Ki67, GFAP, βIII tubulin, vimentin, and Olig2 to quantify proliferation and glia-directed differentiation of neural stem cells in the ASVZ. PCNA was used to label proliferative cells as it is a protein involved in the replication and repair of DNA during the S phase of cell proliferation [71]. GFAP (with no expression of vimentin) and βIII tubulin were used to immunostain mostly the B and A neuroprogenitor cells [31], whereas Olig2 was used to label oligodendrocytes progenitors and mature [32]. We observed a significant decrease of proliferative cells in the ASVZ in the pigs with induced hydrocephalus since the total number of PCNA+ cells was lower compared to the sham control. It is likely that cells of type B, which have been shown to express GFAP without vimentin expression [31] and type A neuroprogenitor cells, can also be affected after the induction of hydrocephalus because their proliferation was found to decrease.

In addition, the total number of Olig2+ cells was reduced in the ASVZ of hydrocephalic pigs. Olig2 is a marker of mature and oligodendrocyte and astrocyte progenitors [32, 33]. The decreased cell proliferation (PCNA+) and Olig2+ cells in the SVZ could be affecting the production of new oligodendrocytes. This reduction of progenitors could be related to the impairments found in the PVWM and ventricular zone, as described in other animal models of hydrocephalus [27, 34, 35, 72]. In addition to the reduction of proliferative cells, the reduction of the area of the SVZ was found to correlate with the increase of the ventricular size. This correlation may be caused by stretch and compression generated by the expansion of the ventricles. The consequences of the reduction of the SVZ are still unclear and require futures studies regarding the different types of SVZ stem cells, their neural fate. Our analyses have been performed in the ASVZ; however, VSVZ may be also affected. The present manuscript describes the first neuropathology characterization of the pig model of hydrocephalus, thus, future studies regarding closer analyses of the VSVZ will be carried out. Nevertheless, the proliferative changes we have observed in the pig model suggest that chronic neurodevelopment impairments may occur.

To put neurodevelopment in the juvenile pig model in perspective clinically, the growth spurt of brain development is similar between domestic pigs and humans [43, 73–75]. In both species, practically all neurogenesis occurs prenatally and the patterns of postnatal neuronal and glial differentiation, myelination, and electrical activity are very similar [76]. At birth, total brain mass is approximately 23% and 25% of adult values in pigs and humans, respectively. Likewise, Conrad et al. [43, 73] report that at 4–12-weeks of age, which is the range of our experiments, brain volume in pigs increases to 50% of adult values, which compares to 2–4 weeks old human brain volume of about 36%; by 21–23 weeks, pig brain volume has increased to 95% of adult levels, compared to 83% in 2-year-old in humans [43]. Therefore, the postnatal time period explored in our pigs studies correlates well with human development [42, 77].

There is a possible role for inflammation in the pathogenesis of acquired hydrocephalus suggested by the increase of pro-inflammatory proteins in the CSF from human and experimental models [12, 16–19]. These findings are consistent with the results shown in the present study, where the levels of the interleukins IL6 and IL8 were significantly increased in the hydrocephalic pigs. IL6 is a pleiotropic cytokine implicated in several nervous system disorders [78]. Although IL6 can have a dual role, it may also has a pro-inflammatory effect [16, 79] as it can be an inflammatory mediator during the acute phase when activating a downstream signaling pathway [78]. IL8 is a leukocyte chemotactic activating cytokine that can be produced by several types of cells after their inflammatory stimulation [79]. Although the levels of the other cytokines and interleukins were not significantly increased after the kaolin injection, possibly due to sample size or the variations observed amongst animals in the same group, trends indicating an increase were detected. In addition, it has been described that pro-inflammatory cytokines and chemokines, including the ones mentioned above, can modify the functions of neural progenitors [80]. For instance, IL6 has been shown to reduce survival, migration, and differentiation of the stem cells in the SVZ; and IL12 may inhibit the proliferation of the neural stem cells [80].

In addition to the CSF pro-inflammatory cytokines and interleukins, the neuropathological examination of brain tissue identified signs of neuro-inflammation such as microglial activation and reactive gliosis associated with hydrocephalus [12]. Periventricular gliosis with an increase of both astrocytes and microglia was detected in the hydrocephalic pigs. Proliferation and hypertrophy of astrocytes and microglial cells have been shown in humans and different animal models of hydrocephalus [9, 10, 27, 35, 39, 40, 55, 81]. Thus, these results indicate that astrocytosis and microgliosis are common pathologies in hydrocephalus, both congenital and kaolin-induced. Some authors have suggested that the increase of microglia can be caused by a global intracranial inflammation generated by the kaolin injection [36]. However, in the present study, the number of these cells was analyzed between 19- and 40-days post-induction and in the PVWM adjacent to lateral ventricles, regions that are remote from the site of the injection and the location of the kaolin obstruction. Furthermore, in pigs injected with kaolin that did not develop ventriculomegaly (kaolin-injected, non-hydrocephalic pigs), microgliosis was not observed in periventricular regions, suggesting that the microglia proliferation was likely more related to hydrocephalus than a global intracranial inflammatory response from kaolin.

We have also observed that gliosis is only statistically significant in the PVWM adjacent to the body of the lateral ventricles. Thus, to know if this increase could be a response or a consequence of elevated ventriculomegaly, correlations with the increase of the total ventricular and body sizes were performed. Although these correlations were not statistically significant, increasing trends were found in the body when compared to the frontal horn.

Although logistically and financially challenging, the use of pigs as an experimental model for hydrocephalus may be useful for investigating the biological mechanisms and sequelae of existing and emerging treatments for hydrocephalus, including CSF shunting and ETV-CPC, which cannot be otherwise investigated in humans or small animal models. One difficulty that is inherent in most experimental models of acquired hydrocephalus is variability in the progression and severity of ventriculomegaly. The high cost of procedures in large animal models and the availability of support staff inhibit frequent post-induction assessments, causing a broad range of survival periods and limited measurements within that time frame. Nevertheless, our previous description of this model indicates that significant ventriculomegaly (i.e., > 2.0 SD from the sam control mean) occurs within the survival periods used in this study. In addition, small sample sizes were included in some of our measurements (e.g., 3D microglia analysis), but we attempted to circumvent this issue by applying rigorous requirements for the inclusion of individual cells.

In conclusion, this study focused on characterizing the changes associated with kaolin induction of hydrocephalus in juvenile pigs. The administration of kaolin produced ventriculomegaly accompanied by ependymal disruption, axon damage in the PVWM, reduced cell proliferation in the SVZ, astroglial and microglial activation, and neuroinflammation. These results suggest that brain development may be affected, but further studies are needed to elucidate the full impact on neuronal and glial differentiation. This model has the potential to be a powerful tool for neurosurgery preclinical studies such as investigating the effects of the ETV-CPC treatments, and for improving the quality of the shunts.

## Supplementary Information

**Additional file 1: Figure S1.** Coronal (A-G) and sagittal (A’-G’) representative MRI images from the seven hydrocephalic pigs used for the tissue analyses. Total ventricular volume (*VV*) and days after the induction of hydrocephalus (*T*) when the MRI was taken for each case are shown.

**Additional file 2: Figure S2.** Percentage of GFAP + and vimentin + cells in the subventricular zone of sham control and hydrocephalic pigs.

**Additional file 3: Figure S3.** Inflammation-ventricular volume correlations. Total ventricular volume versus GFAP + cells from the PVWM adjacent to (A) the frontal horn and (B) body, and Iba1 + cells in the PVWM adjacent to the (C) frontal horn and (D) body correlations in the hydrocephalic pigs. Simple linear regression data (*p* value and R square) are shown for each graph.

**Additional file 4: Figure S4.** Kaolin-injected non-hydrocephalic pig analysis. (**A**) MRI scans before (pre) and after (post) kaolin injections showing no ventriculomegaly after 12–40 days of the induction. (**B**) The number of Iba1 + cells was similar between sham control pigs the kaolin-injected non-hydrocephalic pigs in the PVWM adjacent to the frontal horn and body, suggesting the lack of secondary effects of the kaolin injection.

**Additional file 5: Figure S5.** Time after hydrocephalus induction versus ventricular volume or histology markers. Time after hydrocephalus induction did not correlate with **(A)** total ventricular volume (*black*) or volume of the frontal horn (*yellow*), body (*purple*), atrium (*orange*), or temporal horn (*blue*) of the lateral ventricle; **(B)** ventricular zone disruption adjacent to the frontal horn (*black*) or body (*red*) of the lateral ventricles; **(C)** cell death labeled with cleaved caspase-3 (frontal horn in *black*); **(D)** Olig2 + cells in the PVWM adjacent to the frontal horn in *black* and body in *red*, and the ASVZ (*green*); **(E)** proliferative cells in the ASVZ (PCNA in *black*, PCNA + GFAP in *red*, and Ki67 + βIII tubulin in *green*); **(F)** GFAP + cells from the PVWM adjacent to the frontal horn (*black*) and body (*red*); **(G)** and Iba1 + cells in the PVWM adjacent to the frontal horn (*black*) and body (*red*) in the hydrocephalic pigs. In **(C)**, cell death in the PVWM adjacent to the body of the lateral ventricles may be the only analyzed parameter dependent on time (R^2^ = 0.98, p = 0.0008). Simple linear regression data (*p*-value and R square) are shown for each graph and area/type of cell in the corresponding color.

**Additional file 6: Figure S6.** Analysis of the inflammatory cytokines and interleukins in the cerebrospinal fluid within the same hydrocephalic animals. Analysis of the levels of (**A**) interferon alpha (IFNα), (**B**) interferon gamma (IFNγ), (**C**) interleukin 1 beta (IL1β), (**D**) interleukin 4 (IL4), (**E**) interleukin 6 (IL6), (**F**) interleukin 8 (IL8), (**G**) interleukin 10, (**H**) interleukin 12 (IL12), and (**I**) tumor necrosis factor alpha (TNFα) within the same hydrocephalic pigs (numbered from 1 to 10). Increased trends were found after the induction of hydrocephalus (*post*) compared to CSF before the kaolin administration (*pre*) within each animal.

**Additional file 7: Figure S7.** Correlations between total ventricular volumes and cytokines levels. Simple linear regressions were not significant. Simple linear regression results (*p* value and R square) are shown for each graph.

## Data Availability

All data generated or analyzed during this study are included in this published article and its additional files.

## Abbreviations

ASVZ: Abventricular subventricular zone
CSF: Cerebrospinal fluid
DAPI: 4′,6-Diamidino-2-phenylindole dihydrochloride
ETV-CPC: Endoscopic third ventriculostomy with choroid plexus cauterization
GFAP: Glial fibrillary acidic protein
IFN: Interferon
IL: Interleukin
MRI: Magnetic resonance imaging
PBS: Phosphate buffered saline
PCNA: Proliferating cell nuclear antigen
PVWM: Periventricular white matter
SVZ: Subventricular zone
TNF-α: Tumor necrosis factor alpha
VSVZ: Ventricular subventricular zone
