# Supplementary figures and images for: Acquired hydrocephalus is associated with neuroinflammation, progenitor loss, and cellular changes in the subventricular zone and periventricular white matter
Source: Fluids Barriers CNS. 2022 Feb 22;19:17. doi: 10.1186/s12987-022-00313-3 (PMC8864805; doi:10.1186/s12987-022-00313-3)

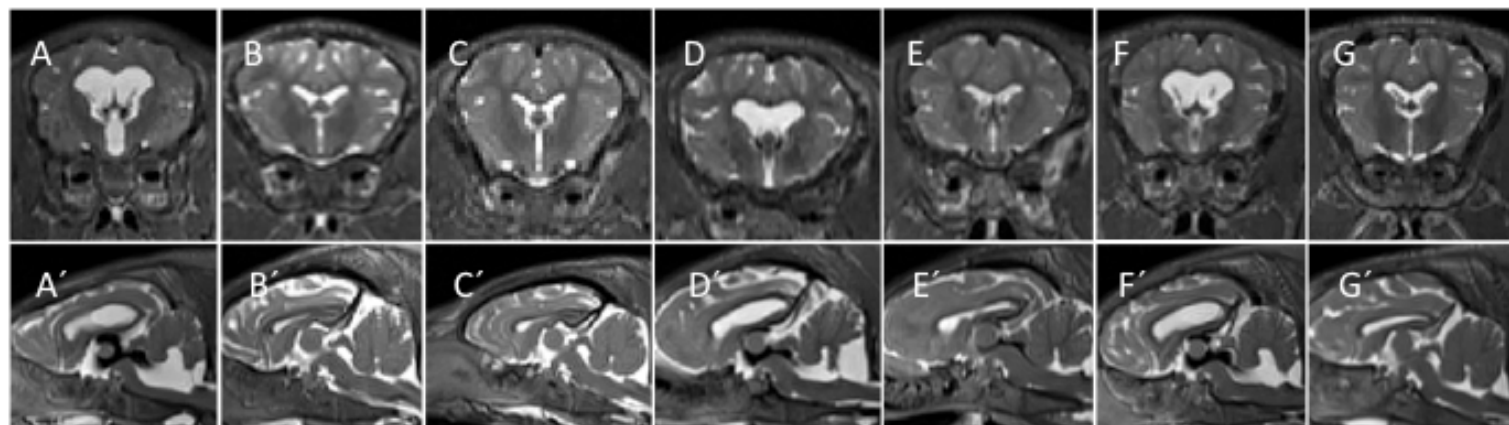

|     |                       |                      |                      |                       |                      |                       |                      |
|-----|-----------------------|----------------------|----------------------|-----------------------|----------------------|-----------------------|----------------------|
| V V | 20824 mm <sup>3</sup> | 3027 mm <sup>3</sup> | 3592 mm <sup>3</sup> | 12802 mm <sup>3</sup> | 4601 mm <sup>3</sup> | 16145 mm <sup>3</sup> | 4391 mm <sup>3</sup> |
| T   | 23 d                  | 28 d                 | 28 d                 | 35 d                  | 19 d                 | 35 d                  | 41 d                 |

Supplement: Supplementary file 1 — Additional file 1: Figure S1. Coronal (A-G) and sagittal (A’-G’) representative MRI images from the seven hydrocephalic pigs used for the tissue analyses. Total ventricular volume (VV) and days after the induction of hydrocephalus (T) when the MRI was taken for each case are shown. [file 12987_2022_313_MOESM1_ESM.pdf]

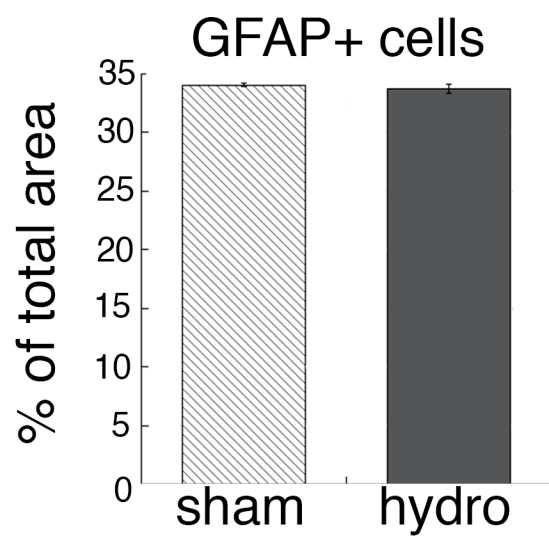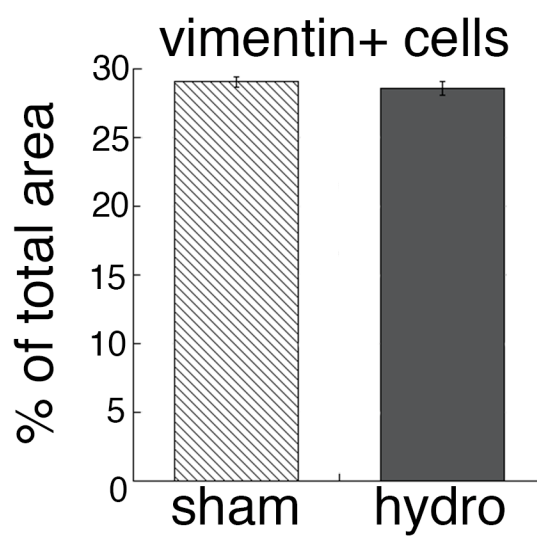

Supplement: Supplementary file 2 — Additional file 2: Figure S2. Percentage of GFAP + and vimentin + cells in the subventricular zone of sham control and hydrocephalic pigs. [file 12987_2022_313_MOESM2_ESM.pdf]

# A kaolin-injected, non-hydrocephalic

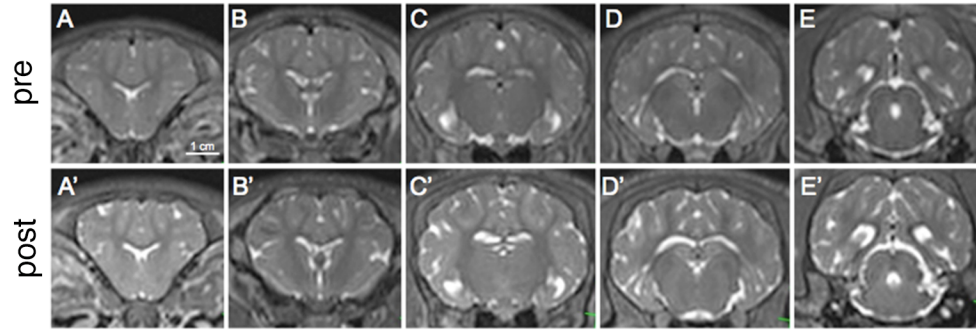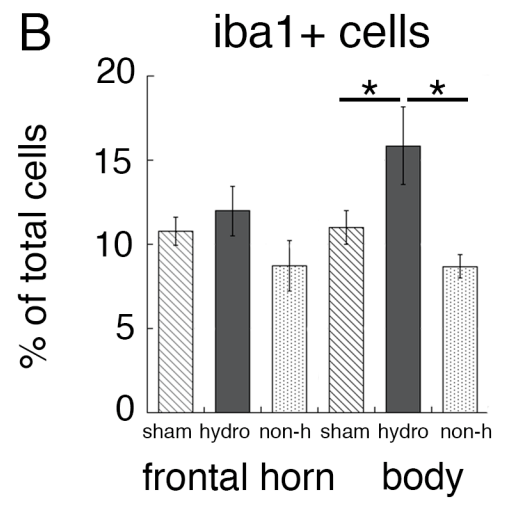

Supplement: Supplementary file 4 — Additional file 4: Figure S4. Kaolin-injected non-hydrocephalic pig analysis. (A) MRI scans before (pre) and after (post) kaolin injections showing no ventriculomegaly after 12–40 days of the induction. (B) The number of Iba1 + cells was similar between sham control pigs the kaolin-injected non-hydrocephalic pigs in the PVWM adjacent to the frontal horn and body, suggesting the lack of secondary effects of the kaolin injection. [file 12987_2022_313_MOESM4_ESM.pdf]

A

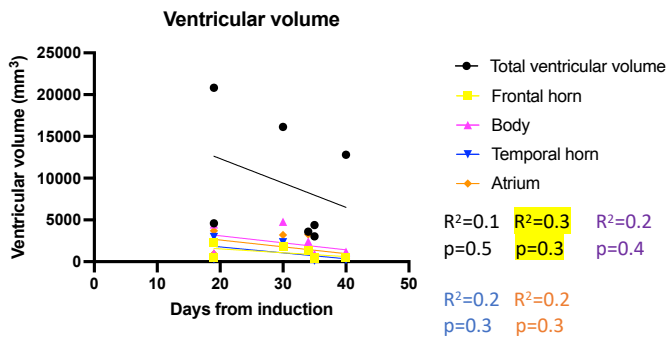

B

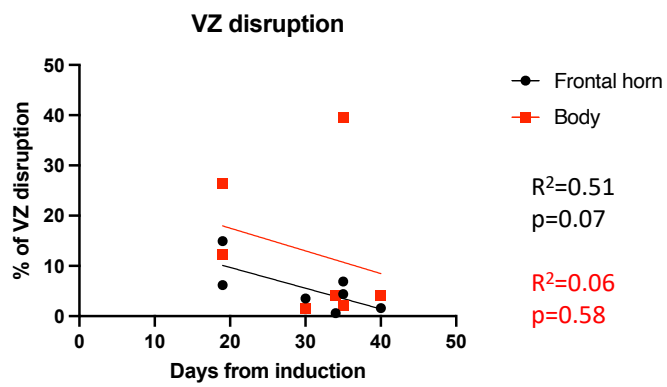

C

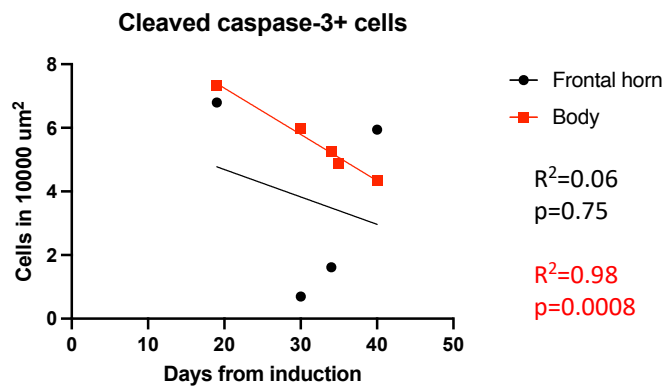

D

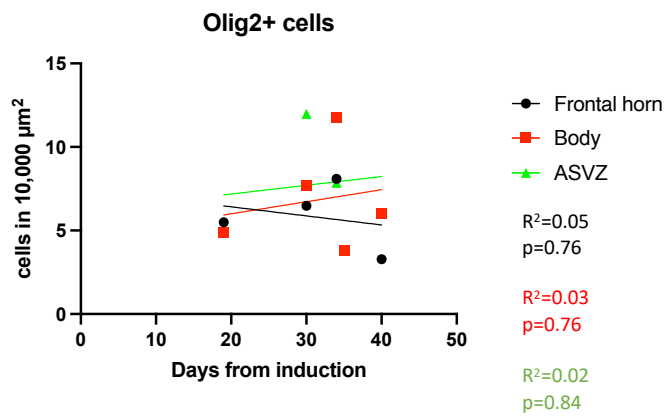

E

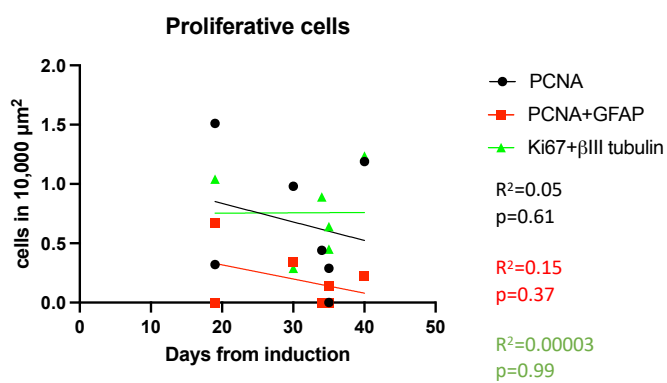

F

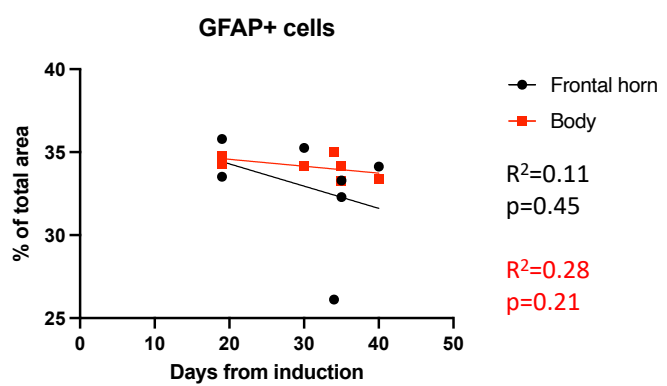

G

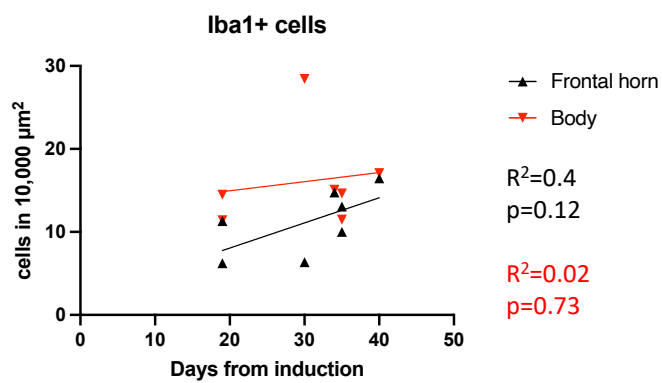

Supplement: Supplementary file 5 — Additional file 5: Figure S5. Time after hydrocephalus induction versus ventricular volume or histology markers. Time after hydrocephalus induction did not correlate with (A) total ventricular volume (black) or volume of the frontal horn (yellow), body (purple), atrium (orange), or temporal horn (blue) of the lateral ventricle; (B) ventricular zone disruption adjacent to the frontal horn (black) or body (red) of the lateral ventricles; (C) cell death labeled with cleaved caspase-3 (frontal horn in black); (D) Olig2 + cells in the PVWM adjacent to the frontal horn in black and body in red, and the ASVZ (green); (E) proliferative cells in the ASVZ (PCNA in black, PCNA + GFAP in red, and Ki67 + βIII tubulin in green); (F) GFAP + cells from the PVWM adjacent to the frontal horn (black) and body (red); (G) and Iba1 + cells in the PVWM adjacent to the frontal horn (black) and body (red) in the hydrocephalic pigs. In (C), cell death in the PVWM adjacent to the body of the lateral ventricles may be the only analyzed parameter dependent on time (R2 = 0.98, p = 0.0008). Simple linear regression data (p-value and R square) are shown for each graph and area/type of cell in the corresponding color. [file 12987_2022_313_MOESM5_ESM.pdf]

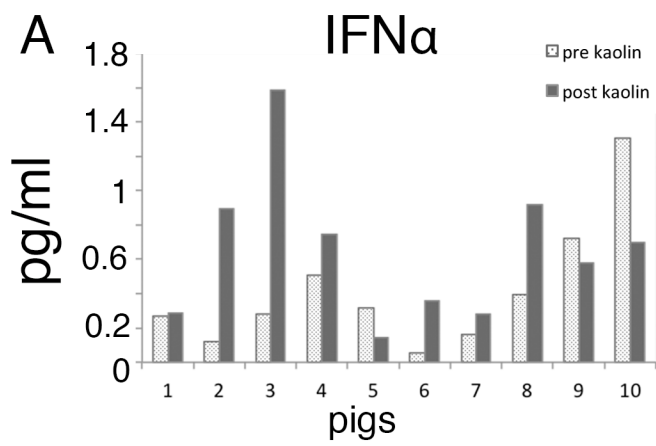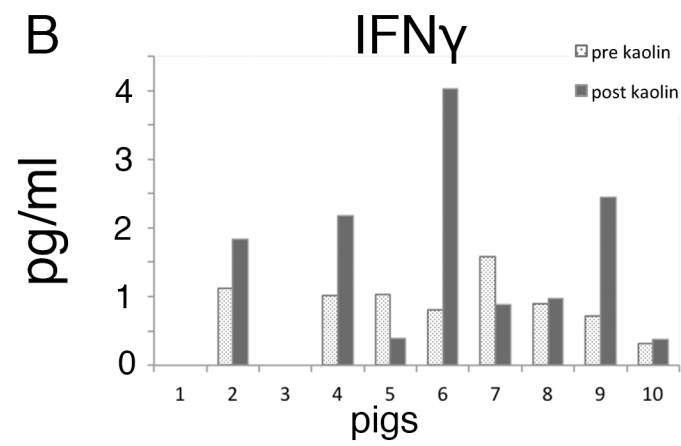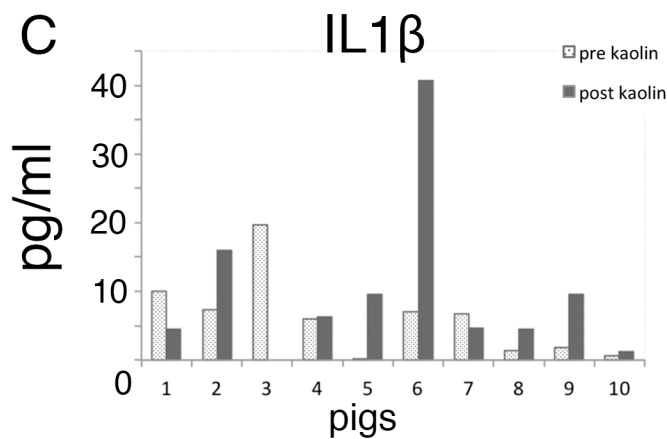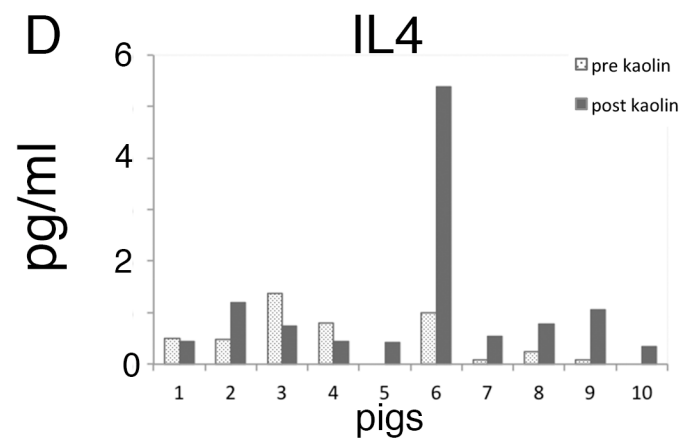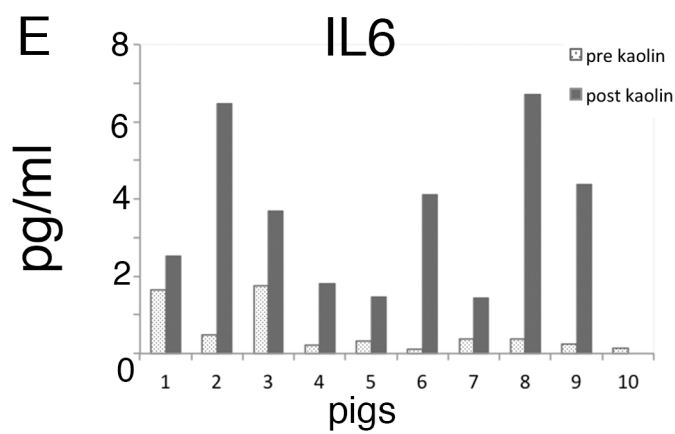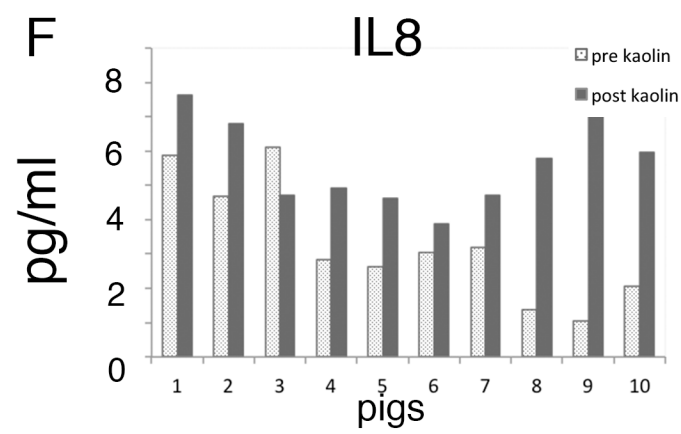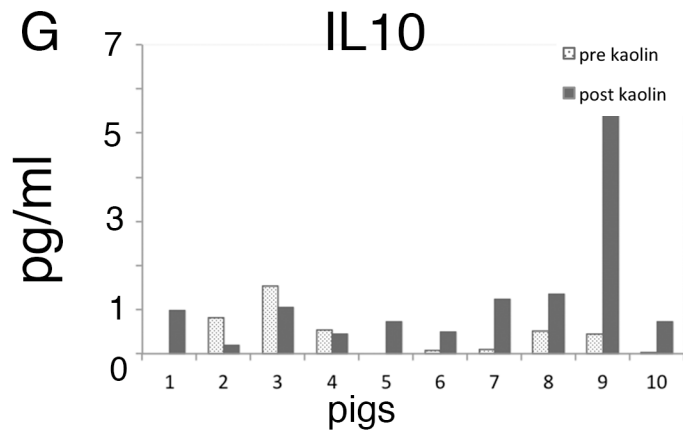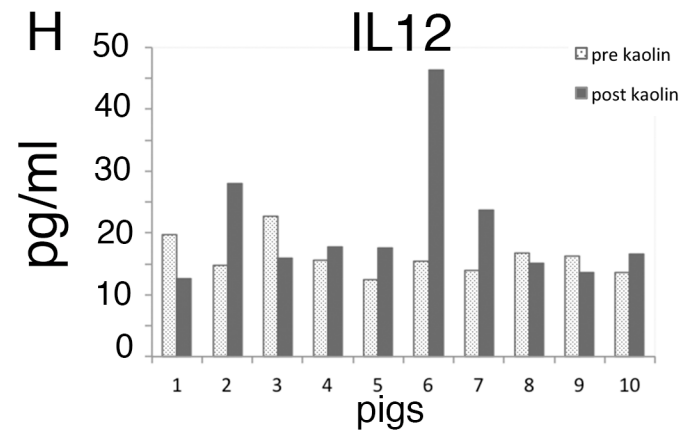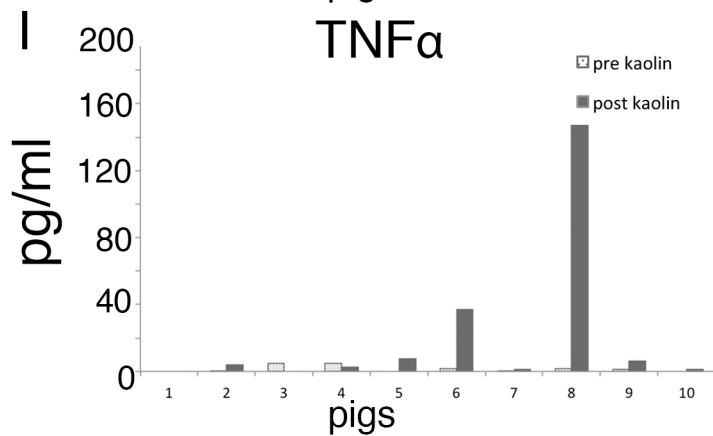

Supplement: Supplementary file 6 — Additional file 6: Figure S6. Analysis of the inflammatory cytokines and interleukins in the cerebrospinal fluid within the same hydrocephalic animals. Analysis of the levels of (A) interferon alpha (IFNα), (B) interferon gamma (IFNγ), (C) interleukin 1 beta (IL1β), (D) interleukin 4 (IL4), (E) interleukin 6 (IL6), (F) interleukin 8 (IL8), (G) interleukin 10, (H) interleukin 12 (IL12), and (I) tumor necrosis factor alpha (TNFα) within the same hydrocephalic pigs (numbered from 1 to 10). Increased trends were found after the induction of hydrocephalus (post) compared to CSF before the kaolin administration (pre) within each animal. [file 12987_2022_313_MOESM6_ESM.pdf]

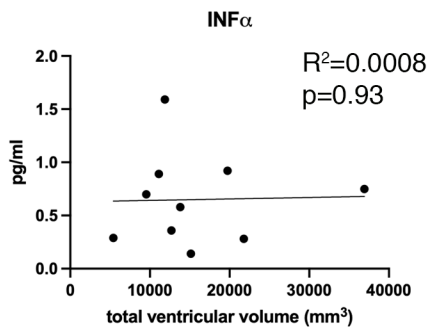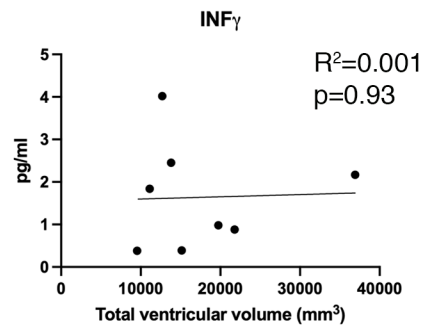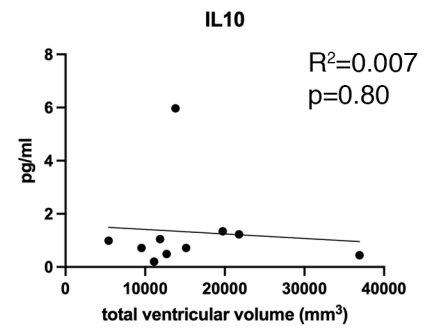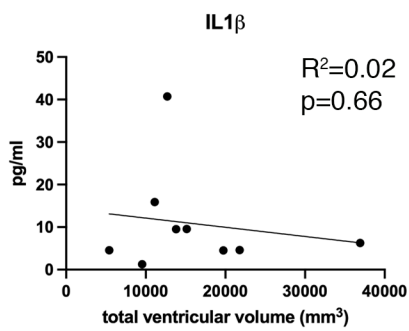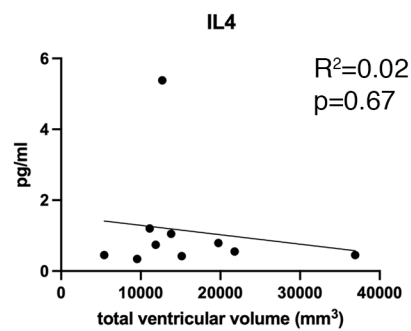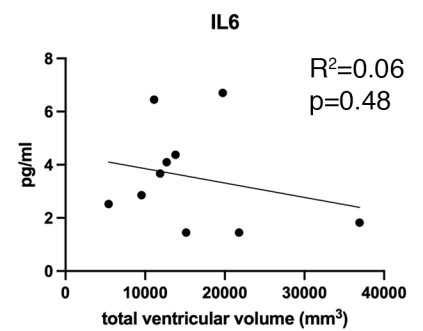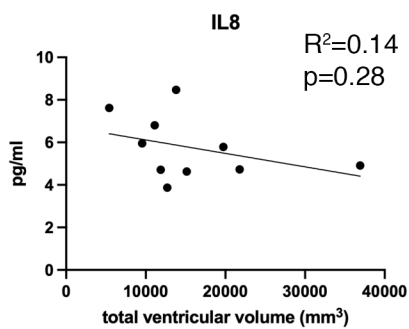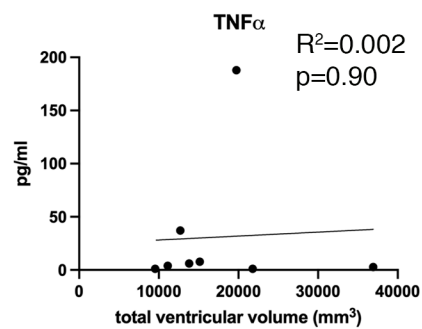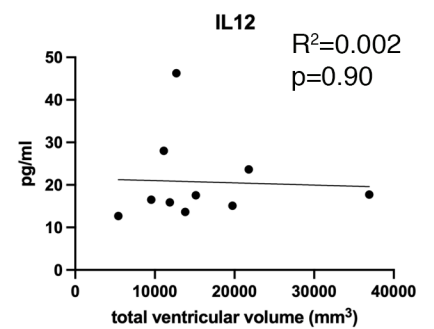

Supplement: Supplementary file 7 — Additional file 7: Figure S7. Correlations between total ventricular volumes and cytokines levels. Simple linear regressions were not significant. Simple linear regression results (p value and R square) are shown for each graph. [file 12987_2022_313_MOESM7_ESM.pdf]
