# Supplementary material for: Acquired hydrocephalus is associated with neuroinflammation, progenitor loss, and cellular changes in the subventricular zone and periventricular white matter
Source: Fluids Barriers CNS. 2022 Feb 22;19:17. doi: 10.1186/s12987-022-00313-3 (PMC8864805; doi:10.1186/s12987-022-00313-3)
Supplement: Supplementary file 3 — Additional file 3: Figure S3. Inflammation-ventricular volume correlations. Total ventricular volume versus GFAP + cells from the PVWM adjacent to (A) the frontal horn and (B) body, and Iba1 + cells in the PVWM adjacent to the (C) frontal horn and (D) body correlations in the hydrocephalic pigs. Simple linear regression data (p value and R square) are shown for each graph. [file 12987_2022_313_MOESM3_ESM.pdf]

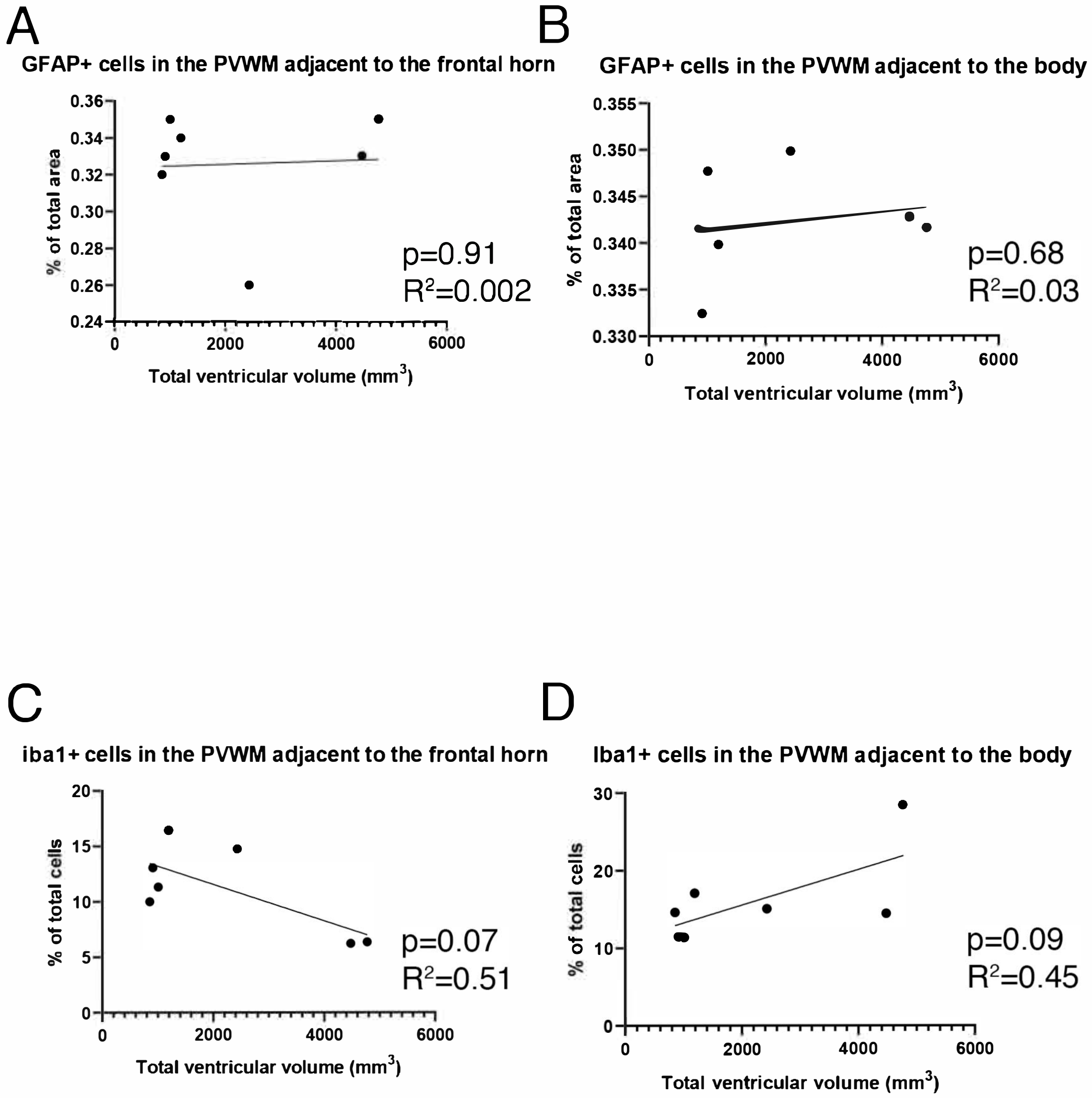
A
GFAP+ cells in the PVWM adjacent to the frontal horn
% of total area
0.36
0.34
0.32
0.30
0.28
0.26
0.24
0
2000
4000
6000
Total ventricular volume (mm3)
p=0.91
R2=0.002
B
GFAP+ cells in the PVWM adjacent to the body
% of total area
0.355
0.350
0.345
0.340
0.335
0.330
0
2000
4000
6000
Total ventricular volume (mm3)
p=0.68
R2=0.03
C
iba1+ cells in the PVWM adjacent to the frontal horn
% of total cells
20
15
10
5
0
0
2000
4000
6000
Total ventricular volume (mm3)
p=0.07
R2=0.51
D
Iba1+ cells in the PVWM adjacent to the body
% of total cells
30
20
10
0
0
2000
4000
6000
Total ventricular volume (mm3)
p=0.09
R2=0.45
